# ACL-ECG: Anatomy-Aware Contrastive Learning for Multi-Lead Electrocardiograms

**DOI:** 10.3390/s26031080

**Published:** 2026-02-06

**Authors:** Wenhan Liu, Zhijing Wu, Zhaohui Yuan

**Affiliations:** School of Information and Software Engineering, East China Jiaotong University, Nanchang 330013, China; whliu@ecjtu.edu.cn (W.L.); 2024218085405008@ecjtu.edu.cn (Z.W.)

**Keywords:** electrocardiogram (ECG), self-supervised learning, contrastive learning, representation learning

## Abstract

Deep learning has achieved impressive progress in automated electrocardiogram (ECG) analysis, yet its performance still relies heavily on large-scale labeled datasets. As ECG annotation requires cardiologists, this process is costly and time-consuming, limiting its scalability in clinical practice. Contrastive learning offers a promising alternative by enabling the extraction of generalizable representations from unlabeled ECG data. In this study, we propose Anatomy-Aware Contrastive Learning for ECG (ACL-ECG), a self-supervised method that incorporates cardiac anatomical relationships into contrastive learning. ACL-ECG employs a physiology-aware augmentation strategy to generate rhythm-preserving augmented views, including random scale cropping, cardiac-cycle masking, and temporal shifting. Furthermore, ECG leads are grouped into four anatomically meaningful regions—anterior, inferior, septal, and lateral—and region-level contrastive objectives are introduced to promote intra-region consistency while enhancing inter-region discriminability. Extensive evaluations of downstream tasks demonstrate that ACL-ECG consistently outperforms state-of-the-art contrastive baselines under linear probing, achieving improvements of up to 1.29% in the area under the receiver operating characteristic curve (AUROC) and 3.57% in the area under the precision–recall curve (AUPRC). Moreover, when fine-tuned using only 10% of labeled data, ACL-ECG attains a performance comparable to fully supervised training, effectively reducing annotation requirements by approximately 5∼8×. Ablation studies further confirm the contributions of both the physiology-aware augmentation strategy and the anatomy-aware contrastive objective. Overall, ACL-ECG enhances representation quality without increasing annotation burden, and provides a promising and anatomy-informed foundation for self-supervised ECG analysis in label-scarce settings.

## 1. Introduction

Cardiovascular diseases (CVDs) are a leading cause of death worldwide, accounting for approximately 33% of global mortality and posing a serious threat to public health [1,2]. Owing to its noninvasiveness, low cost, and ease of use [3], the electrocardiogram (ECG) serves as a primary tool for diagnosing CVDs and provides critical support to cardiologists. By recording the heart’s electrical activity during each cardiac cycle, the ECG captures the physiological states of different cardiac regions from multiple perspectives [4]. The standard 12-lead system, widely used in clinical practice, includes six limb leads (I, II, III, aVR, aVL, aVF) and six precordial leads (V1–V6). The ECG synchronously records the systolic and diastolic processes of the atria and ventricles, producing distinct rhythmic patterns. Its fundamental waveforms comprise the P wave, QRS complex, and T wave: the P wave corresponds to atrial depolarization, the QRS complex reflects ventricular depolarization, and the T wave indicates ventricular repolarization. Cardiovascular pathologies can disrupt these electrophysiological processes, resulting in abnormal waveforms. For example, a flattened or absent P wave may indicate sinus arrest [4], while a widened QRS complex may suggest intraventricular conduction block [4]. Analyzing ECG morphology is therefore critical for assessing cardiac function and diagnosing CVDs. However, significant inter-subject variability and diverse recording conditions make manual interpretation labor-intensive and prone to errors.

In recent years, advances in artificial intelligence have enabled deep learning, particularly supervised learning, to demonstrate considerable potential in automatic ECG recognition and classification. For example, Ribeiro et al. [5] developed a deep neural network (DNN) algorithm capable of identifying six types of cardiac abnormalities from 12-lead ECGs, achieving diagnostic performance surpassing that of cardiologists. Notably, the algorithm exhibited strong generalization to 12-lead ECG data despite being trained on single-lead signals. These results highlight the potential auxiliary diagnostic capabilities of artificial intelligence. Nevertheless, supervised learning heavily relies on large-scale annotated datasets, whereas ECG labeling is costly and labor-intensive [6], creating a bottleneck that limits broader clinical adoption. To address this limitation, unsupervised learning, particularly self-supervised learning, has emerged as a promising research direction. Self-supervised learning generates pseudo-labels from unlabeled data via pretext tasks, enabling models to learn effective feature representations. When applied to ECG analysis, pretrained models can be fine-tuned on downstream tasks to achieve high performance, reducing annotation costs, improving model generalization, and uncovering latent pathological information. Self-supervised methods can be broadly categorized into generative and contrastive approaches [7], distinguished mainly by the design of the pretext task. This study focuses on contrastive learning methods for ECG analysis.

Contrastive learning is a self-supervised approach that leverages intrinsic data properties as supervisory signals to learn discriminative feature representations [8]. Multiple augmented views of the same instance—such as random cropping or Gaussian noise injection—serve as positive samples [9,10], while views from other instances act as negative samples. During training, the encoder is optimized to pull positive pairs closer and push negative pairs apart, ensuring that semantically similar samples are represented closely in the feature space. This enables the model to learn essential data characteristics and generalizable features without manual annotations. Once pretrained, the encoder can be transferred to downstream tasks and used as an effective feature extractor. Contrastive learning has been widely adopted in computer vision, with representative methods including Simple Siamese (SimSiam) [10], Bootstrap Your Own Latent (BYOL) [11], and Simple Framework for Contrastive Learning of Visual Representations (SimCLR) [12]. IThis has recently been applied to ECG analysis, showing promising potential for self-supervised cardiac representation learning.

In ECG analysis, contrastive learning typically adapts computer vision methods to the one-dimensional signal domain by replacing 2D encoders with 1D counterparts and designing ECG-specific data augmentation strategies to construct positive and negative pairs. Key contributions include that of Lan et al. [13], who found distinct representations at both the heartbeat and subject levels for arrhythmia classification using multivariate cardiac signals. Their results demonstrated that the encoder could learn highly informative and generalizable features. Similarly, Wei et al. [14] constructed positive and negative heartbeat pairs using a novel sampling strategy to learn personalized ECG representations that capture patient-specific cardiac conditions, thereby validating the effectiveness of exploiting periodic ECG patterns. Leveraging the unique spatio-temporal properties of ECG, Gopal et al. [15] employed physiological 3D augmentation techniques to generate positive pairs for cardiac pathology classification, demonstrating improved feature learning. Chen et al. [16] proposed a novel Contrastive Learning Framework for Electrocardiogram Arrhythmia Classification (CLECG), combining wavelet transformation with segmented random cropping to construct positive pairs. By applying instance-level contrastive learning on unlabeled data, their method learned generalizable ECG representations for arrhythmia classification, achieving enhanced performance across multiple downstream tasks. Soltanieh et al. [17] systematically analyzed augmentation strategies, further improving downstream performance. Despite these advances, most studies still rely on general image-based contrastive learning frameworks. Liu et al. [18] introduced dense lead contrast to achieve cross-lead feature alignment, enhancing multi-lead ECG representation quality. However, the spatial mapping relationship between leads and cardiac anatomical regions remains underexplored, presenting a key challenge: how to co-design encoder architectures, augmentation strategies, and learning objectives to explicitly incorporate cardiac anatomical priors.

To address this challenge, Anatomy-Aware Contrastive Learning for ECG (ACL-ECG) is proposed as a self-supervised framework that integrates cardiac anatomical priors into representation learning. Independent encoders are assigned to each lead and grouped into clinically meaningful anatomical regions (anterior, inferior, septal, and lateral). Region-aware contrastive objectives are designed to enforce intra-region consistency and inter-region distinctiveness. In addition, a physiology-aware augmentation strategy (Physio-AUG), combining random resized cropping (RRC), cardiac cycle masking (CCM), and temporal shifting (TS), is employed to generate semantically consistent views.

The main contributions of this work are summarized as follows:

1. ACL-ECG Framework: An anatomy-aware contrastive learning framework that models spatial relationships among multi-lead ECGs via independent lead encoders and region-aware objectives.
2. Physio-AUG Strategy: A physiology-inspired augmentation pipeline that preserves cardiac cycle integrity and inter-lead coherence to enhance representation robustness.
3. Comprehensive Validation: Extensive evaluation on three public ECG datasets demonstrates the effectiveness of the proposed method under label-limited settings.

## 2. Materials and Methods

### 2.1. Datasets

The Ningbo First Hospital (NFH) dataset [19] comprises 34,905 12-lead recordings, each 10 s in duration and sampled at 500 Hz. The dataset comprises 56% male and 44% female subjects. Normal recordings account for approximately 20% of the dataset, whereas abnormal recordings comprise the remaining 80%, with the highest prevalence observed in individuals aged 51–80 years. Although clinical diagnostic labels are available in the raw dataset, they were not used for supervised learning in this study. Instead, the dataset served exclusively as an unlabeled source for the self-supervised pretraining, during which all label information was withheld. In preprocessing, recordings with insufficient signal length or severe lead loss were removed, yielding 33,403 high-quality samples for subsequent analysis.

The China Physiological Signal Challenge 2018 (CPSC2018) dataset [20] comprises 6877 12-lead ECG recordings, covering nine rhythm categories: Normal, Atrial Fibrillation (AF), First-degree AV Block (I-AVB), Left Bundle Branch Block (LBBB), Right Bundle Branch Block (RBBB), Premature Atrial Contraction (PAC), Premature Ventricular Contraction (PVC), ST-segment Depression (STD), and ST-segment Elevation (STE). The raw signals were acquired at a sampling rate of 500 Hz, and their durations varied from 6 to 144 s. To standardize the model input, all recordings were adjusted to a fixed length of 10 s: signals longer than 10 s were truncated, while shorter signals were zero-padded at the end. For the classification task, data were partitioned on a subject-wise basis: 20% of subjects were randomly assigned to the test set, and the remaining 80% were split into training and validation sets at a 9:1 ratio. Strict subject independence was ensured across all subsets to prevent overly optimistic performance estimates due to potential data leakage.

The Physikalisch–Technische Bundesanstalt-XL (PTB-XL) dataset [21] comprises 21,837 12-lead ECG recordings from 18,885 subjects. The dataset provides signals sampled at both 100 Hz and 500 Hz; this study uses the 500 Hz version, extracting 10-s segments as model input. According to clinical diagnoses, recordings are grouped into five categories: Normal (NORM), Hypertrophy (HYP), Myocardial Infarction (MI), Conduction Disturbance (CD), and ST/T-wave Changes (STTC). To construct a clear single-label classification task, recordings with multiple diagnostic labels were excluded. This dataset is primarily used to evaluate model performance under standard supervised learning settings. The training, validation, and test splits strictly follow the guidelines provided in the original literature [21] to ensure experimental comparability and reproducibility.

The Chapman dataset [22] is the third 12-lead ECG dataset used for downstream classification in this study and comprises four rhythm categories: Atrial Fibrillation (AFIB), General Supraventricular Tachycardia (GSVT), Sinus Bradycardia (SB), and Sinus Rhythm (SR). All signals were sampled at 500 Hz, with each recording standardized to a fixed length of 10 s. The original dataset contained 10,646 recordings, of which 18 were excluded due to incomplete lead information, yielding a total of 10,628 high-quality recordings for analysis. Data partitioning followed the subject-wise strategy employed in CPSC2018, assigning samples to training, validation, and test sets while ensuring that recordings from the same subject did not appear in multiple subsets. This approach facilitates a realistic assessment of model generalization to unseen subjects.

These four datasets were collected by different institutions, encompassing diverse clinical scenarios and patient populations, thereby enhancing the breadth and representativeness of model validation. Despite the varied sources, all datasets employed subject-wise partitioning to prevent individual information from appearing in both training and testing phases. This approach aligns with the principle of cross-subject validation [23], ensuring that multiple recordings from the same patient are assigned to a single subset, thereby preventing the model from learning patient-specific biases and overestimating performance. Statistical details of each dataset are summarized in Table 1.

### 2.2. Data Preprocessing

Signal preprocessing is a critical prerequisite for ensuring effective feature extraction prior to ECG model construction. The processing pipeline in this study focuses on three key aspects: lead selection, temporal resolution adjustment, and signal amplitude standardization.

In the standard clinical 12-lead system, inherent mathematical redundancies exist among certain leads. According to Einthoven’s limb lead theory [4,24], the information contained in leads I, aVR, aVL, and aVF can be reconstructed via linear combinations of leads II and III, which motivates the use of a reduced lead configuration in this study, as formulated below:

(1) I=II−III

(2) $$\mathrm{aVR}=-\frac{I+\mathrm{II}}{2}$$

(3) $$\mathrm{aVL}=I-\frac{\mathrm{II}}{2}$$

(4) $$\mathrm{aVF}=\mathrm{II}-\frac{I}{2}$$

Given this information redundancy, removing leads I, aVR, aVL, and aVF from the raw data does not result in a substantial loss of diagnostically relevant features. Consequently, limb leads II and III, along with the six precordial leads (V1–V6), are retained to form an eight-channel input configuration. This reduction simplifies the model input structure while preserving physiological integrity.

The raw recordings were originally sampled at 500 Hz for a duration of 10 s, yielding 5000 data points per lead. To ensure compatibility with the input dimensions of the encoder within the network architecture, all signals were resampled to 2048 time steps. This sequence length, which is a power of two, aligns with the multi-stage downsampling design of the convolutional encoder and retains adequate temporal resolution for ECG waveform modeling. Ultimately, each ECG recording is represented as a 2048 × 8 tensor.

Furthermore, significant variations in signal amplitude may exist across different subjects or acquisition devices, often stemming from amplifier gain settings or electrode-skin impedance. To mitigate the interference of such technical variability on model learning, while preserving physiologically meaningful amplitude relationships across leads, Z-score normalization is applied globally across all leads and time points to each eight-lead signal:

(5) $$x^{\prime }=\frac{x-\mu }{\delta }$$

where μ and δ denote the mean and standard deviation of the signal segment across all leads and time points, respectively. Unlike per-lead normalization, which enforces similar amplitude distributions for each lead and may obscure clinically relevant inter-lead amplitude differences, this global normalization strategy removes inter-subject scale variations while retaining relative lead-wise amplitude patterns. This operation helps reduce amplitude offsets and scale variations, harmonizing the statistical distribution of signals from diverse sources, thereby enhancing the stability and generalization ability of model training.

### 2.3. Data Augmentation

Physio-AUG is designed to introduce plausible temporal variability while preserving the fundamental rhythmic structure and inter-lead coherence of ECG signals. The strategy comprises three operations: RRC, CCM, and TS. These augmentations are applied dynamically during training to generate semantically consistent positive views, thereby enabling more effective optimization of the anatomy-aware contrastive learning framework and improving overall model performance. Importantly, the introduction of temporal variability across augmented views is an intentional design choice rather than a preprocessing artifact. By exposing the model to realistic timing perturbations while maintaining semantic consistency, the augmentation strategy encourages the encoder to learn representations that are invariant to temporal misalignment and more robust to acquisition-related variability [25,26].

RRC enhances the model’s robustness to non-standard or partially recorded ECG segments. A subsequence ranging from 80% to 100% of the original signal length is randomly extracted and then resampled via interpolation to the target length of 2048 samples. This process simulates common clinical scenarios such as truncated or incompletely acquired recordings. Importantly, because the cropping window implicitly aligns with whole cardiac cycles or their multiples, the operation avoids disrupting the morphology of individual beats and preserves the essential physiological rhythm. Furthermore, RRC retains different temporal portions of the same ECG across training iterations. As a result, the model is exposed to varying temporal segments of each recording and is encouraged to learn robust and generalizable representations that are not tied to a fixed time window.

CCM is designed to simulate localized signal dropout in physiologically meaningful regions such as the ST segment or T wave. In practice, R-peaks are first detected from the lead V5, and the average RR interval is computed to define the fundamental cycle length. R-peak detection for cardiac-cycle masking is performed using the NeuroKit2 [27] toolkit, which provides a robust and widely validated ECG processing pipeline for reliable R-wave identification across varying signal conditions. Within each cardiac cycle, a random starting position is selected, and a continuous segment spanning 20% of the cycle duration is masked by setting the corresponding time samples in all leads to zero. Because the masked window is strictly constrained within a single cardiac cycle and does not cross the R-peak, CCM introduces localized perturbations while preserving the periodic nature of cardiac electrical activity and the synchronized evolution across leads.

TS simulates temporal alignment discrepancies across different recordings. A shift offset is uniformly sampled within a range of ±80% of the total signal length, with out-of-bounds regions being zero-padded. Although this operation alters the absolute starting position of the signal, the synchronous translation of all leads preserves their relative temporal relationships, ensuring that the augmented views remain consistent with the spatio-temporal patterns of authentic cardiac electrical conduction. The relatively large shift range is intentionally adopted to construct more challenging positive views in the self-supervised setting. Smaller shifts result in highly overlapping signal segments, which may allow the model to minimize the contrastive loss via trivial waveform matching. In contrast, larger shifts with partial zero-padding encourage the encoder to learn shift-invariant and globally meaningful representations, a strategy that has been shown to improve robustness in contrastive representation learning [25].

**Figure 1:**
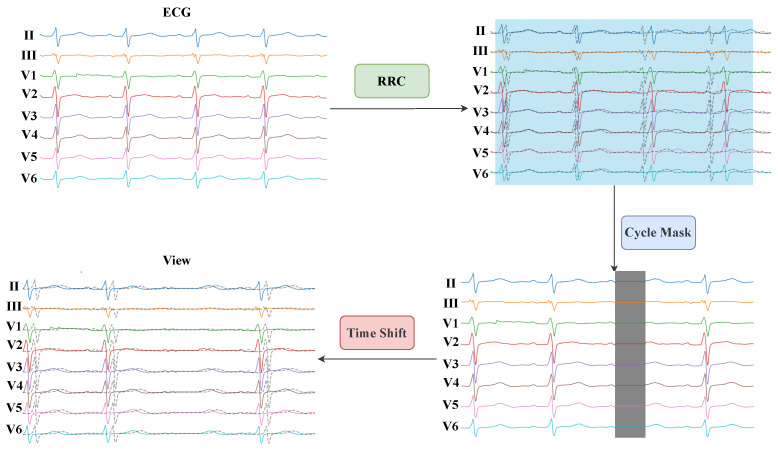
An example of Physio-AUG data augmentation for ECG signals.

The three augmentation strategies described above are applied in combination to generate distinct views of the input samples, which serve as positive pairs for contrastive learning. The entire augmentation pipeline avoids introducing non-physiological artifacts, such as high-frequency noise or baseline wander; instead, it focuses on plausible deformations that preserve the intrinsic structure of cardiac electrical activity. This approach ensures that the generation of pseudo-labels aligns with physiological priors, thereby facilitating the learning of discriminative feature representations. Specific examples of these augmentations are illustrated in Figure 1.

Importantly, all ECG recordings, including low-quality or highly arrhythmic signals (e.g., AF, frequent PVCs, or low-amplitude R waves in V5), are processed using the same augmentation pipeline without rhythm-specific adaptation. This is because the self-supervised pretraining stage relies entirely on unlabeled data, preventing the model from accessing rhythm or quality labels. Retaining such challenging signals can actually enhance the learning of robust and discriminative representations, as they effectively increase the difficulty of the contrastive task, analogous to the use of hard examples in representation learning [25].

### 2.4. Anatomy-Aware Contrastive Learning for Multi-Lead ECGs

The ACL-ECG framework is proposed to integrate cardiac anatomical priors into the representation learning process. By leveraging the anatomical correspondence between ECG leads and specific cardiac regions, leads associated with the same anatomical area are grouped (see Table 2). This grouping is grounded in classical electrocardiographic theory, which establishes well-recognized correspondences between ECG leads and specific myocardial regions (e.g., anterior, inferior, septal, and lateral walls), as extensively documented in the electrocardiology literature [28,29]. Drawing inspiration from the Multi-Branch Network (MBN) architecture [30,31,32,33], independent encoders are configured for each lead to better exploit feature diversity. After feature extraction, anatomically related lead representations are fused through a projector module to learn hierarchical representations with spatial semantics. In downstream tasks, the projector is discarded, and the final prediction is generated by integrating features extracted independently from all leads.

Specifically, data augmentation is first applied to an input sample *x* to generate a view pair $\left(x_{i},x_{j}\right)$. Each view is split along the lead dimension, denoted by $sp\left(x_{i}\right)=\left\{x_{i,1},x_{i,2},\ldots ,x_{i,C}\right\}$ and $sp\left(x_{j}\right)=\left\{x_{j,1},x_{j,2},\ldots ,x_{j,C}\right\}$, where *C* represents the number of leads. In this study, C=8, including the limb leads (II, III) and precordial leads (V1–V6). The lead indices follow the ordering (II, III, V1, V2, V3, V4, V5, V6). Accordingly, *C* independent encoders are employed to process signals originating from the *C* leads separately. Regarding the *c*-th lead, the feature representation pair is derived through the encoder, parameterized by $\theta_{c}$:

(6) $$h_{i,c}=f_{\theta_{c}}\left(x_{i,c}\right),\;h_{j,c}=f_{\theta_{c}}\left(x_{j,c}\right)$$

Guided by anatomical knowledge, the lead-wise features are fused into regional representations. The leads are grouped into four regions: anterior, inferior, septal, and lateral. The regional feature fusion for view $x_{i}$ is formulated as follows:

(7) $$\begin{array}{cc} h_{i}^{\mathrm{ant}} & =h_{i,5}⊕h_{i,6}\;\left(V3,\;V4\right)\\ h_{i}^{\inf} & =h_{i,1}⊕h_{i,2}\;\left(\mathrm{II},\;\mathrm{III}\right)\\ h_{i}^{\mathrm{sep}} & =h_{i,3}⊕h_{i,4}\;\left(V1,\;V2\right)\\ h_{i}^{\mathrm{lat}} & =h_{i,7}⊕h_{i,8}\;\left(V5,\;V6\right) \end{array}$$

where ⊕ denotes the feature concatenation operation. Analogous fusion operations are applied to the representations of the second view $x_{j}$.

For mathematical conciseness in subsequent projections and loss calculations, these D=4 anatomical regions are indexed as d=1,…,D (corresponding to anterior, inferior, septal, and lateral, respectively). The fused regional features are then projected into the feature space via region-specific projectors:

(8) $$\begin{array}{cc} z_{i}^{d} & =g_{\omega_{d}}\left(h_{i}^{d}\right)\\ z_{j}^{d} & =g_{\omega_{d}}\left(h_{j}^{d}\right) \end{array}\;\mathrm{for}\;d=1,\ldots ,D$$

where $\omega_{d}$ represents the parameters of the *d*-th projector. Finally, for the two augmented views of the same sample, two sets of region-level projected features are generated:

(9) $$Z_{i}=\left\{z_{i}^{1},z_{i}^{2},\ldots ,z_{i}^{D}\right\},\;Z_{j}=\left\{z_{j}^{1},z_{j}^{2},\ldots ,z_{j}^{D}\right\}$$

These feature sets are utilized to compute the contrastive loss.

Based on this, a dual-level regional contrastive mechanism is designed, comprising an intra-region contrastive loss ($L_{i,j}^{\mathrm{intra}}$) and an inter-region contrastive loss ($L_{i,j}^{\mathrm{inter}}$). This design aims to separately enhance the model’s ability to capture intra-region consistency and inter-region distinctiveness. Let $z_{i}^{d}$ and $z_{j}^{d^{\prime }}$ represent the projection features of the two augmented views for regions $d,d^{\prime }\in \left\{1,\ldots ,D\right\}$. The specific loss functions are formulated as follows:

(10) $$L_{i,j}^{\mathrm{intra}}=-\sum_{d=1}^{D}\sum_{d^{\prime }=1}^{D}I_{\left[d=d^{\prime }\right]}\log\frac{\exp\left(\frac{s(z_{i}^{d},z_{j}^{d^{\prime }})}{\tau }\right)}{\sum_{k=1}^{2N}I_{\left[k\neq j\right]}\exp\left(\frac{s(z_{i}^{d},z_{k}^{d^{\prime }})}{\tau }\right)}$$

(11) $$L_{i,j}^{\mathrm{inter}}=-\frac{1}{D-1}\sum_{d=1}^{D}\sum_{d^{\prime }=1}^{D}I_{\left[d\neq d^{\prime }\right]}\log\frac{\exp\left(\frac{s(z_{i}^{d},z_{j}^{d^{\prime }})}{\tau }\right)}{\sum_{k=1}^{2N}I_{\left[k\neq j\right]}\exp\left(\frac{s(z_{i}^{d},z_{k}^{d^{\prime }})}{\tau }\right)}$$

where s(·,·) denotes the cosine similarity, τ is the temperature parameter, and *N* is the batch size. The primary distinction between $L_{i,j}^{\mathrm{intra}}$ and $L_{i,j}^{\mathrm{inter}}$ lies in the use of the indicator function $I_{\left[\cdot \right]}$: $I_{\left[d=d^{\prime }\right]}$ considers feature pairs from the same anatomical region, whereas $I_{\left[d\neq d^{\prime }\right]}$ considers pairs from different regions. The scaling factor $\frac{1}{D-1}$ is introduced to normalize the magnitude of $L_{i,j}^{\mathrm{inter}}$. Since $L_{i,j}^{\mathrm{inter}}$ involves summing over D(D−1) combinations of different regions while $L_{i,j}^{\mathrm{intra}}$ involves only *D* combinations, direct accumulation would result in $L_{i,j}^{\mathrm{inter}}$ dominating the total loss, and thereby hindering balanced optimization.

A hyperparameter γ is introduced to balance the intra-region and inter-region losses. The total ACL-ECG loss, $L_{i,j}^{\mathrm{region}}$, is defined as follows:

(12) $$L_{i,j}^{\mathrm{region}}=\gamma \cdot L_{i,j}^{\mathrm{intra}}+\left(1-\gamma \right)\cdot L_{i,j}^{\mathrm{inter}}$$

**Figure 2:**
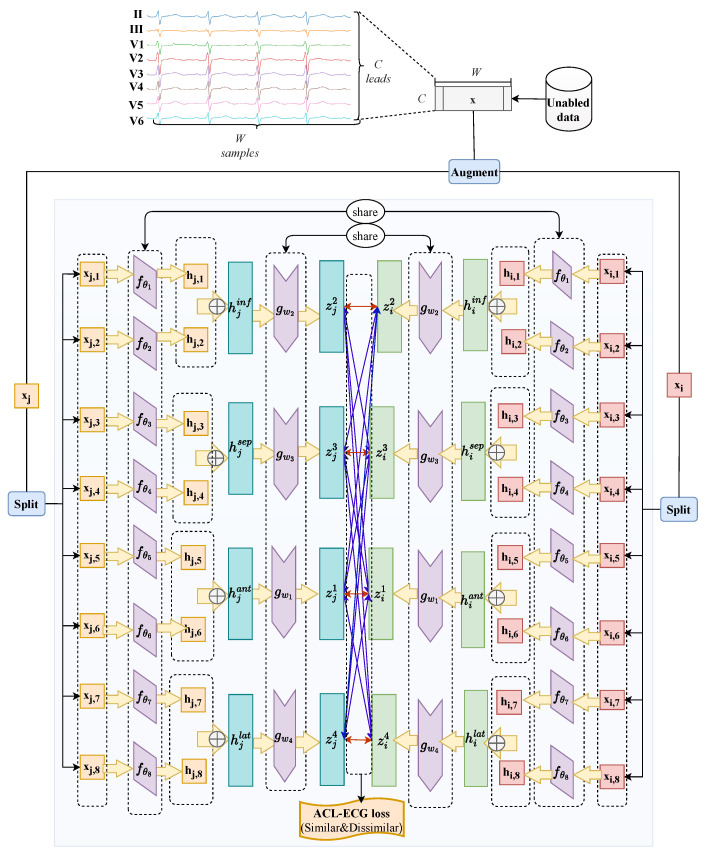
An overview of the proposed ACL-ECG framework.

Consequently, $L_{i,j}^{\mathrm{region}}$ encompasses contrastive losses from pairwise view combinations within the same anatomical region and across different regions, optimizing the model via a loss calculation grounded in cardiac anatomical priors. During pretraining, the total loss $L_{i,j}^{\mathrm{region}}$ is minimized by jointly optimizing the encoder and projector parameters. The detailed procedure is outlined in Algorithm 1. Furthermore, the pretraining process of ACL-ECG is illustrated in Figure 2, which presents the loss calculation based on cardiac anatomical priors.

Upon completion of pretraining, the encoders are applied to subsequent downstream tasks. Within the MBN framework, the lead features output by the encoders are first concatenated into a global feature representation. Subsequently, following standard evaluation practice in contrastive learning, a linear classifier is trained on the global feature representation using cross-entropy loss.

**Algorithm 1**: ACL-ECG
  **Input**: Unlabeled dataset *S*, batch size *N*, temperature τ, lead number *C*, feature

      concatenation ⊕, region number *D*, encoders $f_{\theta_{1}}∼f_{\theta_{C}}$, project head
      $g_{\omega_{1}}∼g_{\omega_{D}}$, data augmentation method *T*, hyperparameter γ

  **Output**: Pretrained encoders 
    
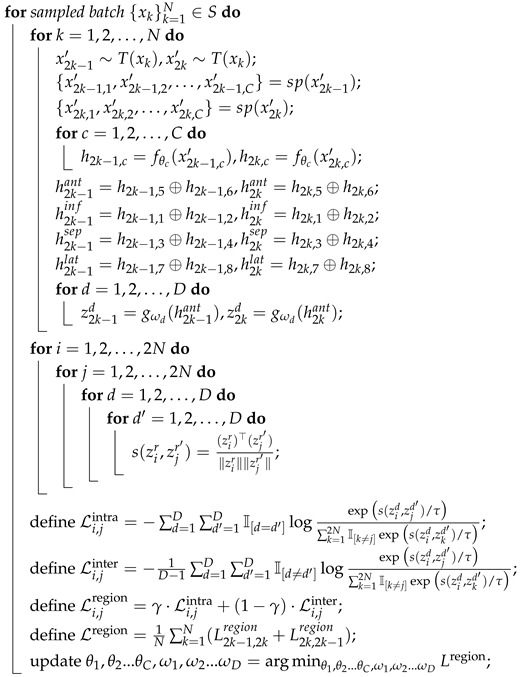



### 2.5. Encoder Architecture

**Figure 3:**
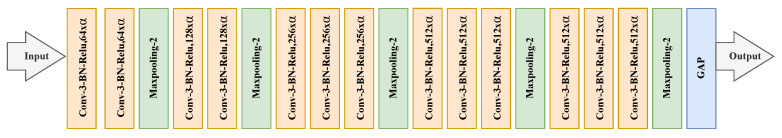
Architecture of the VGGNet-1D encoder.

In this study, a one-dimensional convolutional neural network (1D-CNN) encoder, adopting the architectural style of VGGNet, is employed and designated as VGGNet-1D. The detailed structure of the encoder is illustrated in Figure 3. Notably, within the ACL-ECG framework, a dedicated independent encoder is configured for each lead; consequently, the input for each encoder corresponds to a single-lead ECG signal. To regulate the encoder scale, a width scaling factor of α=0.125 is introduced, ensuring that the feature dimension of the single-lead encoder is equivalent to one-eighth of that of a standard multi-lead encoder.

### 2.6. Experimental Setup and Evaluation

To comprehensively assess the quality of the learned representations, two standard transfer learning evaluation protocols are adopted: linear probing and fine-tuning [34]. In the linear probing setting, the encoder parameters remain frozen, and only a top-layer linear classifier is trained. This evaluates the generalization capacity of the pretrained features. In contrast, fine-tuning updates both the encoder and the classifier, assessing the model’s adaptability to downstream tasks, particularly in semi-supervised scenarios with limited labeled data.

Following this transfer paradigm, the model is first pretrained on the NFH dataset and subsequently applied to downstream classification tasks on the CPSC2018, PTB-XL, and Chapman ECG databases. Comprehensive evaluations are conducted to assess its performance across these datasets. The model is pretrained using the Adam optimizer with a learning rate of 0.001 and a batch size of 64. The temperature parameter is set to τ=0.1. Pretraining is conducted for 200 epochs. For downstream tasks, the Adam optimizer is also employed. Linear probing is trained over 100 epochs at a learning rate of 0.001, while fine-tuning is performed for 100 epochs with a reduced learning rate of 0.0001.

In this study, the Area Under the Receiver Operating Characteristic Curve (AUROC) and the Area Under the Precision–Recall Curve (AUPRC) are adopted as the primary evaluation metrics. For binary classification tasks, samples are labeled as either positive or negative. The classifier outputs a predicted probability indicating the likelihood of a sample belonging to the positive class; based on a predefined threshold (e.g., 0.5), samples can be assigned to positive or negative classes. Based on the predictions and ground truth labels, three fundamental metrics are derived: Recall, Precision, and False Positive Rate (FPR).

Recall, also referred to as the True Positive Rate (TPR), measures the model’s ability to correctly identify positive samples. Precision reflects the proportion of actual positive samples among those predicted as positive. FPR quantifies the proportion of negative samples misclassified as positive. These metrics are mathematically defined as follows:

(13) $$\mathrm{Recall}\;\left(\mathrm{TPR}\right)=\frac{\mathrm{TP}}{\mathrm{TP}+\mathrm{FN}}$$

(14) $$\mathrm{Precision}=\frac{\mathrm{TP}}{\mathrm{TP}+\mathrm{FP}}$$

(15) $$\mathrm{FPR}=\frac{\mathrm{FP}}{\mathrm{FP}+\mathrm{TN}}$$

where TP (True Positives) denotes samples correctly predicted as positive, FP (False Positives) denotes negative samples incorrectly predicted as positive, TN (True Negatives) denotes samples correctly predicted as negative, and FN (False Negatives) denotes positive samples incorrectly predicted as negative.

The values of these metrics vary as a function of the decision threshold. By computing TPR and FPR across different thresholds, the Receiver Operating Characteristic (ROC) curve is constructed, and the AUROC quantifies the model’s overall ability to discriminate between positive and negative classes across all threshold settings. Similarly, by varying the threshold to obtain multiple pairs of Recall and Precision values, the Precision–Recall (PR) curve is plotted, and the corresponding AUPRC provides an aggregate assessment of the model’s performance in identifying positive samples. Since they account for performance over a full range of decision thresholds, AUROC and AUPRC offer a more comprehensive evaluation than metrics computed at a single fixed threshold.

For multi-class classification tasks, a macro-averaging strategy is adopted. Each class is treated as the positive class in turn, while the remaining classes are grouped as the negative classes. The AUROC and AUPRC are then computed separately for each one-vs-rest configuration, and the final performance metrics are obtained by taking the arithmetic mean across all classes. Let *K* denote the total number of classes, and let ${\mathrm{AUROC}}_{k}$ and ${\mathrm{AUPRC}}_{k}$ represent the metrics for the *k*-th class. The overall performance metrics are defined as follows:

(16) $$\mathrm{AUROC}=\frac{1}{K}\sum_{k=1}^{K}{\mathrm{AUROC}}_{k}$$

(17) $$\mathrm{AUPRC}=\frac{1}{K}\sum_{k=1}^{K}{\mathrm{AUPRC}}_{k}$$

This strategy provides an effective and balanced evaluation of model performance in multi-class scenarios.

Mainstream self-supervised learning (SSL) frameworks are adopted as baselines in this study, including SimCLR [12], Momentum Contrast (MoCo) [35], BYOL [11], SimSiam [10], Barlow Twins (BT) [36], Variance–Invariance–Covariance Regularization (VICReg) [37], Dense Lead Contrast (DLC) [18], Direct Lead Assignment (DLA) [38], and Lead Correlation and Decorrelation (LCD) [39]. Except for DLC, all baselines employ a shared-encoder architecture, in which a single encoder with shared weights extracts features from all leads to learn universal ECG representations. For view generation, all baseline methods (unless otherwise specified) utilize Random Resized Crop with Time-Out (RRC-TO) [40] as the data transformation strategy. These methods employ the MBN framework to process multi-lead ECG signals. Specifically, each of the eight leads is fed into a separate encoder branch. For shared-encoder baselines, all branches share identical parameters, whereas DLC assigns an independent encoder to each lead to capture lead-specific features; these encoders are subsequently integrated through the MBN framework for downstream tasks. To ensure a fair comparison, all baseline methods adopt the same model architecture as ACL-ECG and follow consistent experimental protocols, including the optimizer, learning rate schedule, data partitioning, and batch size. Finally, ACL-ECG is evaluated against these baselines on downstream tasks involving the CPSC2018, PTB-XL, and Chapman datasets.

## 3. Results

### 3.1. Implementation and Results

**Figure 4:**
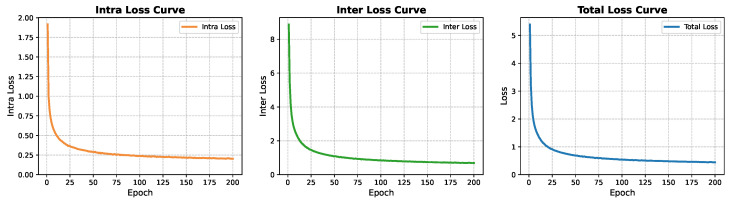
The evolution of loss values during the pretraining phase.

The model is implemented using the PyTorch framework (version 2.5.1). All experiments are conducted on a Windows 10 PC equipped with an Intel^®^ Core^™^ i7-11800H CPU and an NVIDIA GeForce RTX 3050 Ti GPU. Figure 4 illustrates the evolution of loss values during the pretraining phase, demonstrating that all loss components achieve stable convergence as pretraining progresses. As shown in Figure 4, both intra- and inter-region losses decrease steadily during training. Within the first 200 epochs, the intra-region loss is reduced by approximately 86%, while the inter-region loss exhibits a larger reduction of about 91% and converges more rapidly in later stages. All results are averaged over 10 independent runs with different random seeds and reported as mean ± standard deviation.

**Linear probing results.** Table 3, Table 4 and Table 5 present the performance comparison between ACL-ECG and all baseline methods across the three downstream datasets, where ACL-ECG consistently achieves the best results. Specifically, for the CPSC2018 dataset (Table 3), ACL-ECG attains an AUROC of 93.94% and an AUPRC of 74.21%, improving AUROC by 0.95% relative to the strongest baseline (LCD) and surpassing the best-performing baseline (DLC) in AUPRC by 2.96%. On the PTB-XL dataset (Table 4), the model reaches an AUROC of 91.45% and an AUPRC of 71.22%, yielding improvements of 1.29% in AUROC over LCD and 3.57% in AUPRC over DLA. For the Chapman dataset (Table 5), ACL-ECG achieves an AUROC of 99.60% and an AUPRC of 98.69%, with gains of 0.35% and 1.06%, respectively, compared to the best baseline, DLA. Notably, compared with the variant without anatomy-based lead representation merging (DLC), ACL-ECG achieves significantly better performance, highlighting the importance of incorporating lead-level anatomical priors during self-supervised pretraining.

Moreover, when the proposed Physio-AUG strategy in ACL-ECG is replaced with the commonly used RRC-TO augmentation, ACL-ECG still consistently outperforms all competing methods under linear evaluation, indicating that the observed improvements are not solely attributable to the specific augmentation design. ECG-specific SSL methods (ACL-ECG, LCD, DLC, and DLA) consistently outperform general-purpose SSL approaches (e.g., SimCLR, MoCo, BYOL). Across all experiments, ACL-ECG exhibits substantial performance gains over general SSL methods; for instance, on CPSC2018, it surpasses the strongest general baseline, BYOL, by 4.63% in AUROC and 15.28% in AUPRC. In summary, ACL-ECG demonstrates clear advantages in the linear probing setting, consistently outperforming general SSL frameworks and exceeding ECG-specific approaches, thereby highlighting its effectiveness and robustness in ECG representation learning. The statistical significance of ACL-ECG’s improvements is further assessed using DeLong’s test [41], comparing it against the strongest baseline on each dataset. Based on 10 independent runs, improvements exceeding 1% (e.g., on PTB-XL) are statistically significant (*p* < 0.05). In cases with narrower performance gaps (e.g., on the near-saturated Chapman dataset), ACL-ECG achieves results statistically comparable to the strongest baseline (*p* > 0.05 in most runs), confirming its competitiveness even in high-performance regimes. Per-class AUROC and AUPRC under linear probing are reported in Appendix A Table A1. The reported per-class results correspond to a representative run whose overall performance is closest to the macro-averaged metrics.

**Fine-tuning results.** The fine-tuning experiments assess the model’s adaptability to downstream tasks under limited data regimes. Specifically, only 10% of the labeled training samples from the CPSC2018, PTB-XL, and Chapman datasets are used to fine-tune the pretrained encoder, reflecting realistic clinical scenarios where annotated ECG data are costly and scarce. Strong performance in this setting indicates that the model has effectively captured the intrinsic structure of ECG signals during pretraining and exhibits strong few-shot generalization ability. The fine-tuning results, summarized in Table 6, Table 7 and Table 8, show trends largely consistent with the linear probing experiments: ACL-ECG consistently surpasses general-purpose self-supervised learning frameworks and demonstrates superior discriminative feature quality. Furthermore, compared with ECG-specific SSL methods, ACL-ECG remains highly competitive and achieves improvements across most evaluation metrics. In addition, when replacing the proposed Physio-AUG strategy in ACL-ECG with the commonly used RRC-TO augmentation, ACL-ECG still achieves a superior fine-tuning performance to most competing methods, further indicating the robustness of the learned representations to the choice of data augmentation. These findings verify the effectiveness of the proposed architecture in transferring learned ECG representations to downstream classification tasks.In line with the linear probing results, DeLong’s tests show that the fine-tuning improvements were statistically significant (p<0.05) where AUROC gains were substantial. In other cases, ACL-ECG’s performance remains comparable to the top-performing baselines (p>0.05). Per-class AUROC and AUPRC results under fine-tuning are summarized in Appendix A Table A2. The reported per-class results correspond to a representative run whose overall performance is closest to the macro-averaged metrics.

**Computational Cost:** The computational cost of the proposed ACL-ECG framework is reported in terms of training time, GPU memory consumption, and inference latency. The corresponding results are summarized in Table 9. Specifically, the training time is reported for the self-supervised pretraining stage, as well as for linear probing and fine-tuning on the CPSC2018, PTB-XL, and Chapman datasets. Peak GPU memory usage is reported for the pretraining stage, downstream training, and downstream inference. In addition, the inference latency is measured as the average time required to perform inference on a single ECG sample for each downstream task.

**Figure 5:**
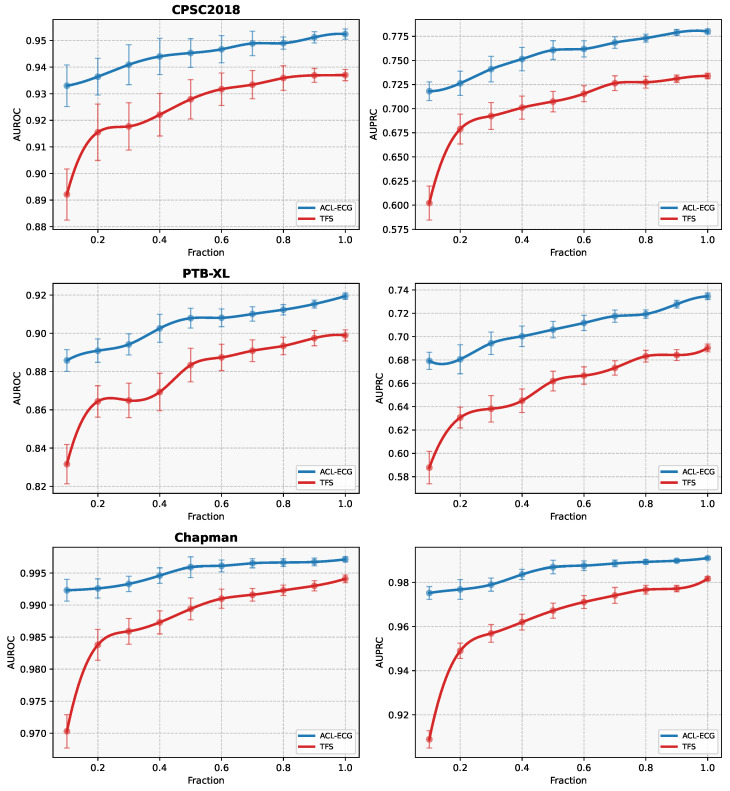
The performance curves of ACL-ECG and the TFS (i.e., trained from scratch) baseline. Error bars indicate the standard deviation over multiple runs with different random seeds. Note that variability is generally higher in low-data regimes and decreases as the amount of labeled data increases.

**Effect of Pretraining:** To further quantify the benefits brought by pretraining, the ACL-ECG model initialized with pretrained weights is compared against a “training-from-scratch (TFS)” baseline. Importantly, the TFS model adopts exactly the same VGGNet-1D encoder architecture as ACL-ECG and is trained using optimization settings identical to those in the fine-tuning stage, including the optimizer, batch size, loss function, and number of training epochs. The only difference between the two models lies in the weight initialization, where TFS is trained from random initialization without pretraining. Figure 5 presents the performance curves of both models under different proportions of labeled data, with error bars representing the standard deviation across multiple independent runs. As the fraction of annotated samples increases, performance improves accordingly, reflecting the positive influence of data scale on model learning capacity. Across all labeling ratios, the pretrained model consistently and substantially surpasses the TFS baseline, with the performance gap being particularly pronounced in low-resource scenarios (e.g., using only 10% or 20% labeled data). Notably, the ACL-ECG model, fine-tuned with only 10% labeled data, achieves a performance comparable to that of the TFS model trained with 50–80% labeled data. This finding indicates that self-supervised pretraining markedly reduces the dependence on manual annotations, enabling strong performance even under very limited supervision and providing a practical solution to the high cost and extended duration associated with medical data annotation.

### 3.2. Ablation Study

This ablation study is designed to investigate the contributions of key modules, operations, and parameters within the ACL-ECG framework. Specifically, the performance of encoders pretrained under different configurations of these components is assessed through linear probing.

**Data Augmentation:** As described in Section 2.3, Physio-AUG constructs semantically consistent positive pairs by introducing time-domain perturbations that adhere to the physiological principles of cardiac electrical activity. It consists of three operations—RRC, CCM, and TS—which respectively simulate clinically relevant scenarios such as incomplete recordings, localized signal dropout, and temporal misalignment. To analyze the contributions of each component of Physio-AUG, Table 10 presents the ablation results obtained by progressively adding individual operations. When only a single augmentation is applied, the model exhibits inferior performance. Overall, the combination of all three operations achieves the best performance.

**Loss Function:** As described in Section 2.4, ACL-ECG partitions ECG leads into four anatomically related groups—anterior, septal, inferior, and lateral—and constructs subspace feature representations for each region. Based on this design, the loss function consists of two components: the intra-region contrastive loss ($L_{i,j}^{\mathrm{intra}}$) and the inter-region contrastive loss ($L_{i,j}^{\mathrm{inter}}$). Accordingly, it is necessary to examine the rationale behind this dual-path loss structure as well as the influence of the balance parameter γ.

**Figure 6:**
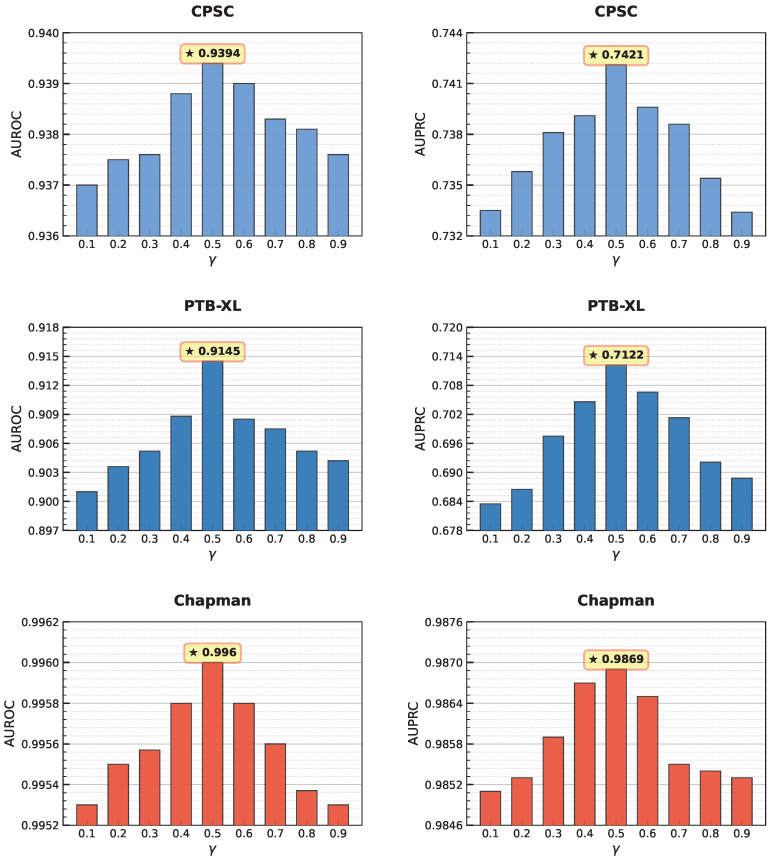
Linear probing results under different values of γ.

Table 11 presents the experimental results obtained under different combinations of loss terms. Using only $L_{i,j}^{\mathrm{intra}}$ or only $L_{i,j}^{\mathrm{inter}}$ results in inferior performance compared with their joint optimization. To further examine the influence of the balance parameter γ, a series of experiments are conducted by sweeping γ within the range [0,1], with the results summarized in Figure 6. Across all downstream tasks, the model achieves the best performance when γ=0.5, indicating that intra-region consistency and inter-region discriminability should be treated with equal importance. Performance degrades when γ deviates from 0.5, suggesting that overemphasizing either loss term is detrimental to global representation learning. These results support the design of a dual-path anatomy-aware contrastive loss.

**Lead Grouping Strategy:** To assess whether the performance gains of ACL-ECG stem from the proposed anatomy-aware lead grouping rather than from arbitrary feature aggregation, we conduct an ablation study comparing the proposed grouping scheme with random lead grouping. Specifically, the eight ECG leads are randomly and uniformly partitioned into four groups, with each group containing two leads. Features within each group are fused and passed through a shared projector to generate region-level embeddings, following the same alignment and contrastive learning objectives as in the anatomy-aware setting. The model is pretrained using the same self-supervised alignment task, and the learned encoders are subsequently transferred to downstream classification tasks under identical evaluation protocols. To mitigate randomness introduced by grouping assignment, the random grouping experiment is repeated 10 times with different random seeds, and the average downstream performance is reported. As summarized in Table 12, random grouping consistently underperforms regarding the proposed anatomy-aware lead grouping across downstream tasks, demonstrating inferior performance and reduced stability.

**Temporal Resolution:** To evaluate the impact of temporal resolution on model performance, a sensitivity analysis is conducted by varying the input length while keeping all other settings fixed. Specifically, input lengths of 512, 1024, 2048, and 4096 time steps are evaluated on the same downstream tasks (see Table 13). The results indicate that 2048 time steps achieve a favorable balance between performance and computational efficiency. Reducing the input length to 512 or 1024 leads to performance degradation, with 512 performing the worst, likely due to the severe loss of fine-grained ECG morphological information, while 1024 provides insufficient temporal resolution to reliably capture clinically important waveform characteristics. Increasing the resolution to 4096 does not yield further performance improvements, but substantially increases memory consumption and computational cost (evaluated on an NVIDIA RTX 3090 with 24 GB memory). Based on these observations, 2048 is adopted as the default input length in all experiments.

## 4. Discussion

### 4.1. Effectiveness of Physio-AUG

The proposed Physio-AUG improves model performance, demonstrating that domain-informed augmentation design is more effective than generic random perturbations. Experimental results show that downstream performance consistently increases when cardiac cycle masking and temporal shifting are progressively incorporated in addition to RRC, suggesting that a single perturbation strategy is insufficient to capture the complex variability of ECG signals.

Specifically, introducing cardiac cycle masking enhances robustness to morphological variations by applying localized perturbations to physiologically critical intervals, such as the ST–T segment. Further incorporating temporal shifting yields additional performance gains, indicating that modeling invariance to temporal misalignment contributes positively to feature consistency. Together, these augmentations play complementary roles in modeling signal completeness, local stability, and temporal invariance.

By simulating clinically relevant conditions such as localized signal dropout and temporal misalignment, Physio-AUG constructs contrastive views that are more challenging yet physiologically coherent, enabling the model to learn robust representations against noise and temporal variability during pretraining. This finding aligns with observations in the image domain, where carefully designed, task-relevant augmentations are critical to the effectiveness of contrastive self-supervised learning [9,42], and further highlights the importance of incorporating physiological priors into augmentation strategies for temporal physiological signals.

### 4.2. Effectiveness of Anatomy-Aware Loss

The anatomy-aware contrastive loss is designed to jointly enforce intra-region consistency and inter-region discriminability, reflecting two complementary requirements for high-quality multi-lead ECG representation learning. In the ablation experiments, variants of ACL-ECG are evaluated using only the intra-region loss $L_{i,j}^{\mathrm{intra}}$, only the inter-region loss $L_{i,j}^{\mathrm{inter}}$, and their weighted combination, to assess the contribution of each component. The experimental results consistently indicate that optimizing either loss term alone leads to inferior performance across all downstream tasks. When only the intra-region loss is applied, the model primarily focuses on enforcing temporal invariance within the same anatomical region, limiting its ability to capture discriminative spatial differences across regions. Conversely, relying solely on the inter-region loss emphasizes regional separability but weakens the modeling of coherent physiological rhythms within each region, resulting in reduced representation stability and generalizability.

In contrast, jointly optimizing both objectives yields the best performance. Across all evaluated tasks, optimal results are achieved when the balancing parameter γ=0.5, indicating that intra-region consistency and inter-region discriminability contribute equally to representation quality. Performance degrades as γ deviates from this value, suggesting that overemphasizing either local invariance or global separability is detrimental to comprehensive representation learning. This dual-path optimization mechanism addresses a key limitation of previous ECG self-supervised learning approaches. Standard contrastive or self-distillation methods, such as BYOL [11] and SimSiam [10], typically treat the multi-lead ECG as a single global representation or process all leads identically, thereby neglecting the intrinsic spatial relationships among leads. Although methods like DLC [18] introduce lead-specific encoders, the anatomical relationships or region-level structure among leads are not explicitly encoded. In contrast, by incorporating clinically grounded anatomical priors (anterior, inferior, septal, and lateral regions) directly into the contrastive objective, ACL-ECG is encouraged to learn representations that are not only discriminative but also physiologically and clinically meaningful.

From a physiological perspective, these results demonstrate that joint optimization preserves consistent morphological patterns within anatomically related leads while maintaining sensitivity to region-specific abnormalities across different cardiac areas. These findings support the hypothesis that integrating domain knowledge into the contrastive learning objective provides a more effective inductive bias than purely data-driven formulations, thereby leading to improved generalization in downstream ECG classification tasks.

### 4.3. Limitations and Future Directions

Despite the strong performance of ACL-ECG, several limitations remain at both the methodological and clinical application levels.

#### 4.3.1. Methodological Limitations

Despite the strong performance of ACL-ECG, several methodological limitations should be acknowledged. First, the proposed Physio-AUG primarily focuses on time-domain transformations and does not explicitly model common non-physiological disturbances such as baseline drift, power-line interference, or electromyographic noise, which are commonly present in real-world ECG recordings. In addition, the RRC augmentation is applied uniformly to all ECG recordings during self-supervised pretraining, without rhythm-specific adaptation. This design choice stems from the fully unlabeled nature of the pretraining data, where rhythm information is not available a priori. As a result, in the presence of highly irregular arrhythmic waveforms, such uniform augmentation may introduce additional difficulty for consistency-based representation learning. Addressing this limitation through rhythm-aware or weakly supervised augmentation strategies represents a promising direction for future work.

Furthermore, while the proposed grouping strategy is physiologically motivated and empirically effective, alternative grouping strategies based on vector angles or spatial projections may provide a more flexible characterization of inter-lead relationships and will be explored in future work. Moreover, although ACL-ECG does not rely on label information during pretraining, the curated nature of the pretraining dataset may still introduce implicit population or case-mix biases, which could affect the transferability of learned representations across diverse clinical settings.

Finally, the current framework is evaluated using an 8-lead ECG configuration derived from the standard 12-lead system. Although the selected leads preserve major spatial information while reducing computational cost, a systematic comparison with native 12-lead input was not conducted. In addition, our evaluation is limited to curated, relatively clean public datasets (e.g., CPSC2018, Chapman) and, in the case of PTB-XL, to single-label recordings following standard benchmarking protocols. Real-world clinical settings often involve multi-label pathologies, unfiltered signals with high-amplitude artifacts, and variable-length monitoring such as Holter ECGs. The robustness and generalization of ACL-ECG under these more complex and noisy conditions have yet to be validated, and future work will focus on extending this evaluation to multi-label, uncurated, and real-world clinical datasets.

#### 4.3.2. Clinical Application-Level Limitations

From a clinical application perspective, the current study focuses on an 8-lead ECG configuration derived from the standard 12-lead system. This reduced-lead representation preserves most diagnostically relevant information due to the inherent redundancy among limb leads and has therefore been widely adopted in prior ECG learning studies. However, it may be suboptimal for highly specialized clinical tasks that rely on the full 12-lead configuration. In particular, applications such as fine-grained myocardial infarction localization or low-voltage ECG analysis may benefit from the additional spatial information provided by the complete set of precordial leads, which is not fully captured in the eight-lead setting. Consequently, while the proposed framework demonstrates strong generalization across multiple downstream tasks, its clinical applicability to lead-sensitive diagnostic scenarios remains to be further validated. Extending anatomy-aware self-supervised learning to full 12-lead ECGs and systematically evaluating its impact on localization-sensitive tasks therefore constitutes an important direction for future work.

## 5. Conclusions

This study introduces ACL-ECG, a self-supervised learning framework designed for ECG analysis. Existing deep learning approaches rely heavily on large-scale labeled datasets, while annotating ECG signals is costly and labor-intensive. ACL-ECG addresses this limitation by leveraging unlabeled ECG data and incorporating both physiological and anatomical priors during pretraining. Its core mechanisms include Physio-AUG and an anatomy-aware contrastive loss that jointly model intra-region consistency and inter-region discriminability. Experimental results demonstrate that ACL-ECG achieves competitive or superior performance compared with mainstream baselines, while improving representation quality. It learns physiologically meaningful features from unlabeled data and maintains a performance comparable to fully supervised models under the same experimental settings, even when fine-tuned with only 10% labeled data. Ablation studies further confirm that each component of Physio-AUG and the dual-path contrastive loss contributes positively to representation learning.

## Figures and Tables

**Table 1:** Statistical distribution of the ECG databases.

	Pretraining			
NFH	33,403			
	Class	Training	Validation	Test
CPSC2018	Normal	1213	197	365
	AF	1289	168	342
	I-AVB	889	101	251
	L-BBB	243	33	56
	RBBB	1964	292	589
	PAC	864	130	274
	PVC	1084	146	308
	STD	1148	178	345
	STE	264	58	68
	Total	8958	1303	2598
PTB-XL	NORM	7254	916	913
	HYP	1353	172	184
	MI	416	64	56
	CD	2048	234	256
	STTC	1907	256	243
	Total	12,978	1642	1652
Chapman	AFIB	1583	186	449
	GSVT	1639	186	472
	SB	2804	189	769
	SR	1625	315	436
	Total	7651	161	2126

**Table 2:** Correspondence between ECG leads and cardiac anatomical regions.

Cardiac Region	Corresponding Leads
Anterior	V3 V4
Septal	V1 V2
Inferior	II III
Lateral	V5 V6

**Table 3:** Linear probing performances of ACL-ECG and the baselines using the CPSC2018 database.

Method	AUROC	AUPRC
SimCLR	0.8494 ± 0.0117	0.5065 ± 0.0165
MoCo	0.8776 ± 0.0071	0.5677 ± 0.0098
BYOL	0.8931 ± 0.0077	0.5893 ± 0.0106
SimSiam	0.7685 ± 0.0089	0.3701 ± 0.0136
BT	0.8315 ± 0.0078	0.4798 ± 0.0098
VICReg	0.8322 ± 0.0103	0.4745 ± 0.0145
DLC	0.9282 ± 0.0063	0.7125 ± 0.0108
DLA	0.9299 ± 0.0024	0.7091 ± 0.0047
LCD	0.9267 ± 0.0085	0.7067 ± 0.0126
ACL-ECG(RRC-TO)	0.9338 ± 0.0061	0.7229 ± 0.0083
ACL-ECG	**0.9394 ± 0.0070**	**0.7421 ± 0.0086**

Bold values are the best results; underlined values are the second-best results.

**Table 4:** Linear probing performances of ACL-ECG and the baselines using the PTB-XL database.

Method	AUROC	AUPRC
SimCLR	0.8792 ± 0.0077	0.6412 ± 0.0084
MoCo	0.8682 ± 0.0059	0.6253 ± 0.0069
BYOL	0.8812 ± 0.0046	0.6542 ± 0.0056
SimSiam	0.7469 ± 0.0119	0.3999 ± 0.0225
BT	0.8677 ± 0.0048	0.6065 ± 0.0052
VICReg	0.8665 ± 0.0124	0.6121 ± 0.0141
DLC	0.8902 ± 0.0044	0.6736 ± 0.0051
DLA	0.9014 ± 0.0059	0.6765 ± 0.0122
LCD	0.9016 ± 0.0029	0.6716 ± 0.0033
ACL-ECG(RRC-TO)	0.9056 ± 0.0029	0.6874 ± 0.0037
ACL-ECG	**0.9145 ± 0.0031**	**0.7122 ± 0.0049**

Bold values are the best results; underlined values are the second-best results.

**Table 5:** Linear probing performances of ACL-ECG and the baselines using the Chapman database.

Method	AUROC	AUPRC
SimCLR	0.9599 ± 0.0038	0.8796 ± 0.0045
MoCo	0.9622 ± 0.0025	0.8884 ± 0.0059
BYOL	0.9777 ± 0.0019	0.9193 ± 0.0021
SimSiam	0.8461 ± 0.0156	0.6228 ± 0.0196
BT	0.9363 ± 0.0053	0.8139 ± 0.0076
VICReg	0.9385 ± 0.0056	0.8179 ± 0.0074
DLC	0.9912 ± 0.0010	0.9701 ± 0.0011
DLA	0.9925 ± 0.0012	0.9763 ± 0.0015
LCD	0.9876 ± 0.0008	0.9634 ± 0.0018
ACL-ECG(RRC-TO)	0.9934 ± 0.0008	0.9804 ± 0.0010
ACL-ECG	**0.9960 ± 0.0006**	**0.9869 ± 0.0012**

Bold values are the best results; underlined values are the second-best results.

**Table 6:** Fine-tuning performances of ACL-ECG and the baselines using the CPSC2018 database.

Method	AUROC	AUPRC
SimCLR	0.9002 ± 0.0111	0.6301 ± 0.0268
MoCo	0.8965 ± 0.0108	0.6321 ± 0.0284
BYOL	0.9115 ± 0.0051	0.6565 ± 0.0173
SimSiam	0.8178 ± 0.0128	0.3934 ± 0.0298
BT	0.8933 ± 0.0082	0.6206 ± 0.0164
VICReg	0.9073 ± 0.0096	0.6233 ± 0.0158
DLC	0.9188 ± 0.0074	0.6902 ± 0.0131
DLA	**0.9336 ± 0.0082**	0.7114 ± 0.0155
LCD	0.9179 ± 0.0105	0.6836 ± 0.0172
ACL-ECG(RRC-TO)	0.9328 ± 0.0070	0.7156 ± 0.0102
ACL-ECG	0.9330 ± 0.0078	**0.7181 ± 0.0096**

Bold values are the best results; underlined values are the second-best results.

**Table 7:** Fine-tuning performances of ACL-ECG and the baselines using the PTB-XL database.

Method	AUROC	AUPRC
SimCLR	0.8577 ± 0.0108	0.6344 ± 0.0126
MoCo	0.8635 ± 0.0081	0.6412 ± 0.0139
BYOL	0.8715 ± 0.0069	0.6409 ± 0.0111
SimSiam	0.7458 ± 0.0144	0.4425 ± 0.0265
BT	0.8437 ± 0.0055	0.6208 ± 0.0087
VICReg	0.8704 ± 0.0079	0.6277 ± 0.0083
DLC	0.8691 ± 0.0111	0.6291 ± 0.0153
DLA	0.8756 ± 0.0040	0.6558 ± 0.0049
LCD	0.8639 ± 0.0088	0.6509 ± 0.0096
ACL-ECG(RRC-TO)	0.8776 ± 0.0048	0.6618 ± 0.0059
ACL-ECG	**0.8858 ± 0.0057**	**0.6792 ± 0.0073**

Bold values are the best results; underlined values are the second-best results.

**Table 8:** Fine-tuning performances of ACL-ECG and the baselines using the Chapman database.

Method	AUROC	AUPRC
SimCLR	0.9801 ± 0.0066	0.9352 ± 0.0076
MoCo	0.9810 ± 0.0075	0.9409 ± 0.0081
BYOL	0.9830 ± 0.0039	0.9532 ± 0.0046
SimSiam	0.9372 ± 0.0178	0.8049 ± 0.0226
BT	0.9814 ± 0.0069	0.9433 ± 0.0088
VICReg	0.9788 ± 0.0063	0.9401 ± 0.0079
DLC	0.9885 ± 0.0017	0.9672 ± 0.0027
DLA	0.9916 ± 0.0019	0.9724 ± 0.0039
LCD	0.9886 ± 0.0150	0.9617 ± 0.0036
ACL-ECG(RRC-TO)	0.9920 ± 0.0013	0.9738 ± 0.0024
ACL-ECG	**0.9923 ± 0.0017**	**0.9752 ± 0.0029**

Bold values are the best results; underlined values are the second-best results.

**Table 9:** Computational cost of ACL-ECG across different training and inference stages. Time: training time. Train Mem: Peak GPU memory usage during training. Infer Mem: Peak GPU memory usage during inference. Latency: Average per-sample inference latency.

Phase	Dataset	Time (s)	Train Mem (MB)	Infer Mem (MB)	Latency (ms)
Pretraining	NFH	27,826.07	1935.40		
Linear Probing	CPSC2018	18.07	55.10	30.30	13.86
	PTB-XL	27.54	55.10	30.20	14.47
	Chapman	15.88	55.10	32.90	13.18
Fine-tuning	CPSC2018	6103.30	1938.10	30.30	13.86
	PTB-XL	6543.80	1938.00	30.20	14.47
	Chapman	5621.73	1938.00	32.90	13.18

**Table 10:** Linear probing results based on different augmentations. RRC: Random Resized Crop; CCM: Cardiac Cycle Masking; TS: Time Shifting. * indicates the use of the corresponding augmentation.

Dataset	RRC	CCM	TS	AUROC	AUPRC
CPSC2018	*	*	*	**0.9394**	**0.7421**
	*	*		0.9379	0.7375
	*		*	0.9374	0.7367
		*	*	0.9334	0.7272
	*			0.9340	0.7305
		*		0.8971	0.6070
			*	0.9327	0.7201
PTB-XL	*	*	*	**0.9145**	**0.7122**
	*	*		0.9022	0.6906
	*		*	0.8967	0.6710
		*	*	0.8955	0.6713
	*			0.8861	0.6639
		*		0.8464	0.5685
			*	0.8935	0.6613
Chapman	*	*	*	**0.9960**	**0.9869**
	*	*		0.9954	0.9851
	*		*	0.9956	0.9868
		*	*	0.9957	0.9862
	*			0.9953	0.9857
		*		0.9889	0.9658
			*	0.9956	0.9858

Bold values are the best results.

**Table 11:** Linear probing results based on different combinations of losses. * indicates the use of the corresponding loss term.

Dataset	$L_{i,j}^{\mathrm{intra}}$	$L_{i,j}^{\mathrm{inter}}$	AUROC	AUPRC
CPSC2018	*	*	**0.9394**	**0.7421**
	*		0.8825	0.5659
		*	0.9340	0.7313
PTB-XL	*	*	**0.9145**	**0.7122**
	*		0.8862	0.6509
		*	0.9009	0.6822
Chapman	*	*	**0.9960**	**0.9869**
	*		0.9857	0.9557
		*	0.9952	0.9847

Bold values are the best results.

**Table 12:** Performance comparison between anatomy-aware lead grouping and random grouping strategies. RG: Random Grouping; AAG: Anatomy-Aware Grouping.

	CPSC2018		PTB-XL		Chapman	
Grouping Strategy	AUROC	AUPRC	AUROC	AUPRC	AUROC	AUPRC
RG	0.9361	0.7286	0.9042	0.6788	0.9912	0.9698
AAG	**0.9394**	**0.7421**	**0.9145**	**0.7122**	**0.9960**	**0.9869**

Bold values are the best results.

**Table 13:** Performance comparison under different temporal input lengths.

	CPSC2018		PTB-XL		Chapman	
Beat Length	AUROC	AUPRC	AUROC	AUPRC	AUROC	AUPRC
512	0.8166	0.6077	0.7923	0.5423	0.9769	0.9461
1024	0.8992	0.6596	0.8688	0.6159	0.9883	0.9672
2048	**0.9394**	**0.7421**	**0.9145**	**0.7122**	**0.9960**	**0.9869**
4096	0.9302	0.7337	0.9087	0.6997	0.9935	0.9831

Bold values are the best results.

## Data Availability

The NFH, PTB-XL, CPSC2018, and Chapman databases are publicly available at: https://physionet.org/content/challenge-2021/1.0.3/training/ningbo/#files-panel (accessed on 14 January 2026), https://physionet.org/content/ptb-xl/1.0.3/ (accessed on 15 December 2025), http://2018.icbeb.org/Challenge.html (accessed on 14 January 2026), and https://figshare.com/collections/ChapmanECG/4560497/2 (accessed on 14 January 2026).

## Appendix A. Per-Class Performances of ACL-ECG

**Table A1:** Per-class AUROC and AUPRC scores for all datasets under the linear probing.

Database	Class	AUROC	AUPRC
CPSC2018	Normal	0.9287	0.7223
	AF	0.9842	0.9351
	I-AVB	0.9616	0.7812
	LBBB	0.9982	0.8312
	RBBB	0.9718	0.9168
	PAC	0.8421	0.4853
	PVC	0.8852	0.6687
	STD	0.9537	0.7784
	STE	0.9273	0.5559
	Average	0.9392	0.7417
PTB-XL	NORM	0.9185	0.9123
	CD	0.9072	0.7127
	HYP	0.9238	0.7684
	MI	0.8818	0.3911
	STTC	0.9427	0.7725
	Average	0.9148	0.7114
Chapman	AFIB	0.9981	0.9853
	GSVT	0.9929	0.9832
	SB	0.9992	0.9912
	SR	0.9934	0.9895
	Average	0.9959	0.9873

**Table A2:** Per-class AUROC and AUPRC scores for all datasets under fine-tuning.

Database	Class	AUROC	AUPRC
CPSC2018	Normal	0.9427	0.6823
	AF	0.9812	0.9427
	I-AVB	0.9883	0.8821
	LBBB	0.9921	0.8554
	RBBB	0.9754	0.9572
	PAC	0.7741	0.2953
	PVC	0.8667	0.5966
	STD	0.9582	0.7783
	STE	0.9201	0.4775
	Average	0.9332	0.7186
PTB-XL	NORM	0.9127	0.9356
	CD	0.9223	0.7227
	HYP	0.9063	0.6983
	MI	0.7258	0.1255
	STTC	0.9599	0.9123
	Average	0.8854	0.6789
Chapman	AFIB	0.9901	0.9392
	GSVT	0.9902	0.9766
	SB	0.9976	0.9961
	SR	0.991	0.9882
	Average	0.9922	0.9750
